# Characterization of lab-based swarms of *Anopheles gambiae* mosquitoes using 3D-video tracking

**DOI:** 10.1038/s41598-023-34842-0

**Published:** 2023-05-30

**Authors:** Andrea Cavagna, Irene Giardina, Michela Anna Gucciardino, Gloria Iacomelli, Max Lombardi, Stefania Melillo, Giulia Monacchia, Leonardo Parisi, Matthew J. Peirce, Roberta Spaccapelo

**Affiliations:** 1 CNR-ISC (National Research Council — Institute for Complex Systems), Rome, Italy; 2 Physics Department University Sapienza, Rome, Italy; 3 INFN Unità Di Roma 1, Rome, Italy; 4 Department of Medicine and Surgery, University of Perugia, Perugia, Italy; 5 Centro Universitario Di Ricerca Sulla Genomica Funzionale (C.U.R.Ge.F), CIRM Italian Malaria Network, University of Perugia, Perugia, Italy

**Keywords:** Biological physics, Computational biophysics, Behavioural ecology

## Abstract

Mosquito copulation is a crucial determinant of its capacity to transmit malaria-causing *Plasmodium* parasites as well as underpinning several highly-anticipated vector control methodologies such as gene drive and sterile insect technique. For the anopheline mosquitoes responsible for African malaria transmission, mating takes place within crepuscular male swarms which females enter solely to mate. However, the mechanisms that regulate swarm structure or that govern mate choice remain opaque. We used 3D-video tracking approaches and computer vision algorithms developed for the study of other complex biological systems to document swarming behavior of a lab-adapted *Anopheles gambiae* line in a lab-based setting. By reconstructing trajectories of individual mosquitoes lasting up to 15.88 s, in swarms containing upwards of 200 participants, we documented swarm-like behavior in both males and females. In single sex swarms, encounters between individuals were fleeting (< 0.75 s). By contrast, in mixed swarms, we were able to detect 79 ‘brief encounters’ (> 0.75 s; < 2.5 s) and 17 longer-lived encounters (> 2.5 s). We also documented several examples of apparent male-male mating competition. These findings represent the first steps towards a more detailed and quantitative description of swarming and courtship behavior in one of the most important vectors of malaria.

## Introduction

The ability of *Anopheles gambiae* females to transmit the malaria-causing parasite, *Plasmodium falciparum*, is heavily dependent on the mosquito’s high reproductive rate which supports the large mosquito population required to sustain transmission^1^. Indeed, progress over the last 20 years in reducing the burden of malaria mortality and morbidity has been largely driven by measures to reduce the vector population^2^. Despite this progress, malaria continues to impose a huge global public health cost; in 2021 there were 241 million malaria infections causing 627,000 deaths^3^. Worryingly, in recent years, the downward trend in case numbers has stalled and even reversed as mosquitoes develop resistance to the insecticides used in treated bed nets and indoor residual spraying programs; the mainstays of hitherto effective vector control efforts^4^.

The consequent imperative to develop effective new tools for vector control has placed a renewed focus on mosquito reproduction and mating behavior as a key pinch point in controlling mosquito populations^5–8^. Mating is fundamental to the efficacy of some of the most keenly anticipated and innovative new methods of vector control including gene drive^9,10^, sterile insect technique (SIT)^11,12^ and the vertical transmission of endosymbiont bacteria such as *Wolbachia*^13^. However, the safe and effective deployment of these approaches may be hindered by the significant knowledge gaps in our understanding of mosquito mating behavior^5,7,14^.

The mosquitoes responsible for African malaria transmission mate largely, if not exclusively^15^, in the context of male swarms that form at dusk^16^. Females enter the swarm solely to mate, an event that takes place on the wing and lasts as little as 15 seconds^17^. Huge strides have been made in recent years in our understanding of the characteristics of male swarms and their species-specific variation^18–24^. However, fundamental questions remain.

Specifically, the complex, close-range behaviors that take place within the swarm remain incompletely understood^5,7,14^. For example, despite significant efforts^25–27^ much remains to be learned about the collaborative male-male behaviors that make the swarm a cohesive structure. Whether and how these differ in the intensely competitive setting of locating and mating with a female (the primary purpose of the swarm), is also unclear. Other unresolved questions relate to the relative importance of male-male competition and female mate choice in deciding male mating success^5^. Some have suggested that the rapid rate of coupling observed in field swarms precludes meaningful female choice^16,17^ implying male mating success is essentially random^28^ or defined entirely by between-male competition. However, lab observations have documented active female rejection behaviors such as kicking off unwanted males and male avoidance behaviors^17^, suggesting a degree of female choice. In the case that female choice is a factor, what does male–female ‘courtship’ behavior look like and how is it mediated?

One possibility is that acoustic signals may have a role^29,30^. A striking example is ‘harmonic convergence’, whereby a prospective couple may actively ‘tune’ their fundamental wingbeat frequencies such that the harmonic frequencies (multiples of the respective fundamentals) converge. In *Aedes aegypti* this acoustic interaction is more frequently observed in the minority of couples who go on to copulate successfully^31,32^. At the same time, studies in *An. gambiae* confirm the importance of different wingbeat frequencies in distinguishing males from females, but suggest that harmonic convergence is not predictive of mating success but rather, a random consequence of males adopting a wingbeat frequency that optimizes female audibility by ‘tuning out’ the preponderance of male background noise^33^. Thus, the manner in which *An. gambiae* males and females identify and choose one another has yet to be fully explained.

Answering these fundamental questions and understanding such complex behaviors is impossible if we cannot observe and document them, a task complicated by their fast-moving nature and complexity (swarms can contain hundreds or even thousands of males). In other complex groupings such as flocks of starlings^34,35^ and midge swarms^36^ stereoscopic 3D tracking has proved capable not only of providing objective quantitative descriptions of these complex aggregations but has also yielded useful biological insight. This approach uses multiple, high resolution video cameras to gather footage of the group in question. Using computer vision algorithms, the path of each individual in a swarm can then be reconstructed in time and 3D space^37^. Similar approaches have been used to study mosquito swarms in the field^38,39^. These studies employed a low frame rate (25fps) meaning reconstructed trajectories were only defined up to a time resolution of 0.04 s, and swarms of moderate size (a few dozen mosquitoes), which may limit statistical rigour. Despite these technical challenges, this work yielded new insight into male swarm dynamics^25^ as well as documenting and describing apparent mating couples within the swarm^27,39^. However, if we aspire to define differences in swarming behavior, for example between transgenic and wild-type mosquitoes, a more controlled environment is required.

Therefore, with the aim of providing a quantitative characterization of individual and group behavior of ‘reference’ mosquitoes, we recreated swarms of the lab-adapted *An. gambiae* strain G3, under laboratory conditions. In this controlled environment, where sex separation is possible, we could study both single-sex and mixed-sex swarms. Through the analysis of 3D reconstructed trajectories of swarms of up to 200 individuals, we provide novel insight into the complex structure of the swarms and quantitatively prove differences in female and male exploration strategies. In the context of mixed-sex swarms, we document several different types of apparent mating encounters and examples of male-male competition. This is the first time, to our knowledge, that the apparently complicated motion of mosquitoes has been described at a quantitative level, and represents a novel step forward in the development of swarming models and in the definition of benchmarks for mosquito motion.

## Results

Using the laboratory-controlled setup described in ‘Methods’ section, we aimed to: 1—characterize the mosquito participation ratio in single-sex swarms; 2—characterize mosquito kinematics in single-sex swarms, to provide a quantitative benchmark against which other mosquito lines could be compared; 3—detect and describe male–female mating-associated encounters in mixed-sex swarms. At the same time, we also aimed to validate our experimental setup, showing that the characteristics we found in lab-adapted swarms were in agreement with previously reported field studies and observations.

### Single-sex swarming participation

The swarming behavior we observed using the set up described in Methods, was very much as described previously^40^; minutes after the daylight illumination was extinguished, swarms began to form over the ground marker. This behavior was repeatable for a single mosquito generation observed over multiple days, from one generation to the next and, moreover, was seen in both males and females. Once formed, swarms maintained a stable position for as long as the artificial twilight was on (30/40 min). This allowed the swarming behavior of each mosquito generation to be documented in multiple sequential video recordings (acquisitions). Acquisitions lasted 15.88 s each (the maximum recording time of our camera system working at 170fps), and were interspersed with a 7–8-min pause to allow data downloading.

**Figure 1:**
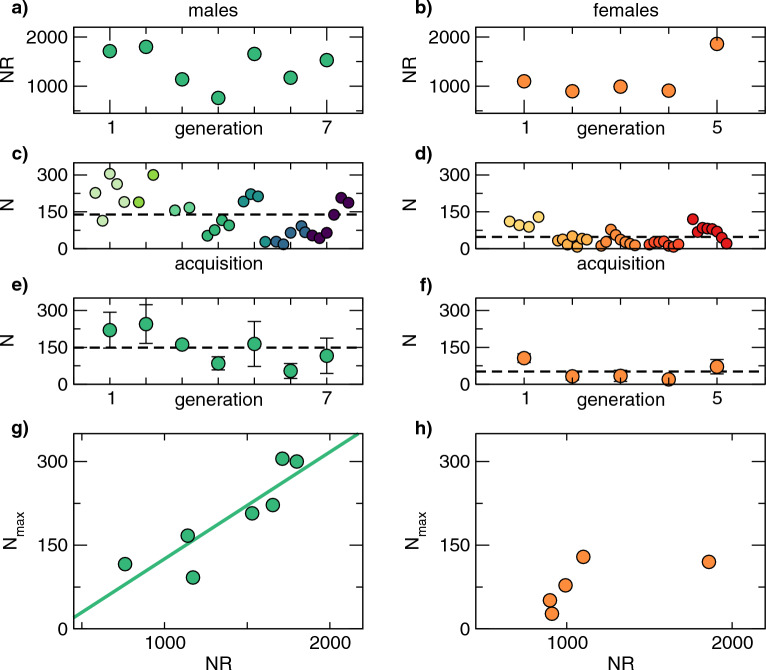
Characteristics of lab-based *An. gambiae* swarms. (**a**) and (**b**) the number, *NR*, of mosquitoes released into the cage, for each generation of males (left) and females (right). *NR* is in the same range, between approx. 800 and 2000, in both cases, but is less variable for females. (**c**) and (**d**) The number, *N*, of mosquitoes participating in 28 acquisitions of single-sex male swarms (left column) and 34 single-sex female swarms (right column) are shown. Different colours refer to different generations. Male swarms are on average larger (mean ± standard deviation, 138 ± 84.6) than female swarms (48 ± 34.4). (**e**) and (**f**) *N* is averaged over all acquisitions of the same generation, left column for male swarms and right column for females. Data represents mean ± standard deviation. (**g**) and (**h**) *Nmax*, the maximum number of swarming mosquitoes across all acquisitions of the same generation, as a function of *NR*. In males *Nmax* depends linearly on *NR* (Pearson coefficient 0.88 – *p* value 0.005). The slope of the linear fit, equal to 0.19, represents the typical participation ratio in male swarms. In females the linearity between *Nmax* and *NR* is not evident.

We characterized mosquito swarm participation analyzing single-sex swarms belonging to different generations, see Methods. As depicted in Fig. 1 panels a and b, the number *NR* of released mosquitoes in the 7 generations of males varied between 760 and 1800, while in the 5 generations of females *NR* ranged between 900 and 1900 but with limited variability (in 4 of the 5 generations *NR* is close to 1000). For each generation we analyzed multiple acquisitions, for a total of 28 acquisitions of males and 34 acquisitions of females. We automatically counted the number *N* of mosquitoes participating in the swarm for each acquisition, see Methods. As shown in Fig. 1, the number of participating mosquitoes varied significantly between individual acquisitions of the same generation (panels c, d) as well as from one generation to the next (panels e, f). Despite this variability, the mean number of participants was larger in males (*N* between 18 and 305) than in females (*N* between 18 and 129).

We wanted to understand whether the number of swarm-participating mosquitoes was related to the number of mosquitoes (*NR*) released into the cage. Therefore, we expressed, for each generation, a number, *Nmax,* representing how many mosquitoes reacted to our swarming stimuli even for a short time (see ‘Methods’ section for precise definition). As shown in Fig. 1, panel g, in males Nmax was linearly related to *NR* (Pearson coefficient 0.88, *p* value 0.005), and the linear fit gives a participation ratio, defined as *Nmax/NR*, of 0.19. For females the situation was more complex. Only a small portion of the released females organize in a swarm, with *Nmax* always smaller than 150, which seems to indicate that the number of participating females does not depend on *NR*, as suggested by the literature^22^. Note that the analyzed data exhibit low variability in *NR*, which in 4 of the 5 generations is close to 1000. Therefore, it may also be the case that we would need a wider range of *NR* to detect a dependence between *Nmax* and *NR*. In either case, the existence or lack of such a dependency, the participation ratio in females is lower than in males.

**Figure 2:**
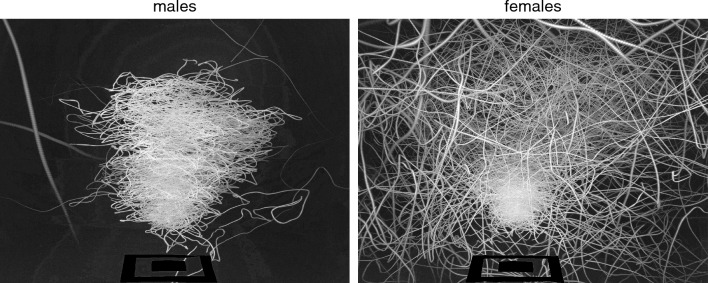
Swarm structure in single-sex male and female swarms. The superimposition of 2700 images from one of the cameras, clearly shows a gross difference in swarm structure. Male swarms (left panel) are stable and have a roughly cylindrical form, while non-participating males remain stationary on the cage walls. In contrast, female swarms (right panel) comprise two distinct zones, a dense, relatively ordered aggregation near the ground marker, and a second zone, at some distance from the ground marker, characterized by much more disordered female motion.

In terms of swarm structure, females display gross differences from males, as judged by superimposing 2700 frames of swarming footage from a single camera (Fig. 2). Male swarms tend to have a stable cylindrical or barrel-shape structure, and males not participating in the swarm generally lie still on the walls of the cage. Female behavior is quite distinct. Swarms are small and take the form of a dense central ‘knot’ near the ground marker, around which is a much looser, more chaotic zone in which females, either fail to join the swarm or enter the swarm and rapidly leave. Together these data document swarming behavior in both male and female members of a lab-adapted *An. gambiae* strain as well as identifying some gender specificities in that behavior.

### Characteristics of 3D motion during swarming

**Figure 3:**
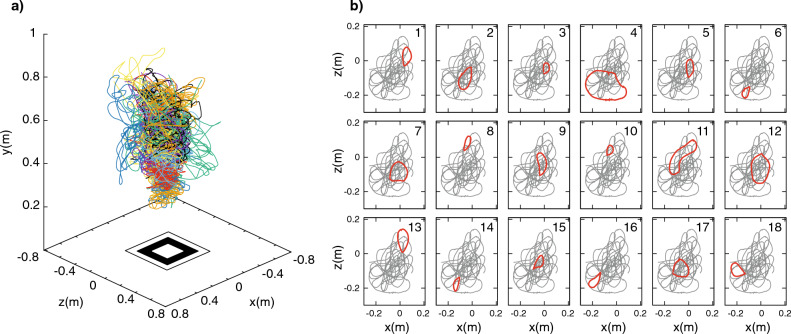
Characteristic circular movements in the horizontal plane. (**a**) The 3D reconstructed trajectories of a single-sex male swarm. Different colours represent different mosquitoes. (**b**) The trajectory of a single individual in the horizontal plane, as it would be seen from above the swarm. Mosquitoes perform consecutive pseudo-circular motions, highlighted in red, superimposed on the complete trajectory of the same individual, shown in light grey. Numbers in the top right corner of each sub-panel represent the temporal order of the ring-like movements.

At first glance, individual mosquito trajectories in a swarm intertwine in complicated ways, see Fig. 3a. Mosquitoes seem to explore the volume occupied by the swarm following random paths. But a deeper analysis of 3D reconstructed trajectories reveals a structured motion, characterized by repetitive flattened helical flight paths that appear as ring-like patterns in the horizontal plane, parallel to the ground. In Fig. 3b, we show the trajectory of a single mosquito as it would be seen from above the swarm, i.e. its projection on the horizontal plane, highlighting its consecutive, ring-like movements in a time series, moving from top left to bottom right.

**Figure 4:**
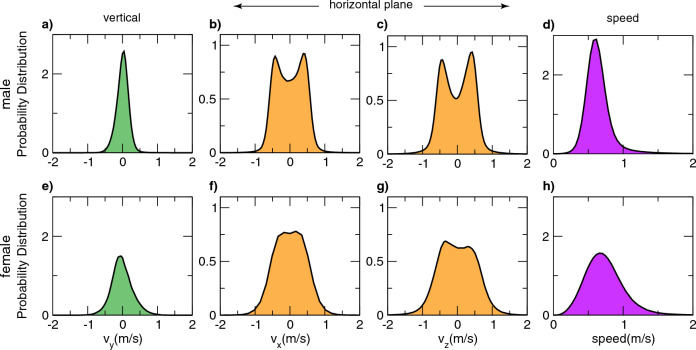
Differences in 3D velocities in male and female swarms. Graphs represent the probability distribution of the three components of the velocity and of the speed, for 19 male swarms and 11 female swarms. In both males (top row) and females (bottom row), we found mean values of *v*_*x*_, *v*_*y*_ and *v*_*z*_ close to 0, confirming the stable position in time of swarms at a group level. Top row: males. The narrow distribution of *v*_*y*_, i.e. the velocity component parallel to the direction of gravity, together with the double-peaked distributions of *v*_*x*_ and *v*_*z*_, reveals that motion mainly occurs on the *xz*-plane. The distributions of *v*_*x*_ and *v*_*z*_, with the two peaks at ± 0.5 m/s, further indicate the lack of any favoured direction of flight on the *xz*-plane, but rather, in the swarm all directions on the horizontal plane are explored with the same probability, i.e. a change in the *v*_*x*_, *v*_*z*_ reference frame would display the same double-peaked distributions. Bottom row: females. Female motion is qualitatively distinct, with the distributions of the three components of the velocity and of the overall speed wider than in males. Females are more prone to move in the direction of gravity, with a *v*_*y*_ distribution larger than males (std = 0.38 m/s against the std = 0.18 m/s that we found in males), and with the double peak of the distributions of *v*_*x*_ and *v*_*z*_ being barely visible.

Are these ring-like patterns on the horizontal plane unique to the individual in Fig. 3b or representative of mosquito movements more broadly during swarming? To answer this question, from mosquitoes’ 3D trajectories we computed the velocity vectors and the probability distribution of their vertical component *v*_*y*_, i.e. the velocity component parallel to the direction of gravity, and of the two horizontal components, *v*_*x*_ and *v*_*z*_, as shown in Fig. 4. We found mean values of *v*_*x*_, *v*_*y*_ and *v*_*z*_ close to 0, as expected given the stable position of the swarms at a group level: single mosquitoes display complicated trajectories with an average speed of 0.59 m/s (see Fig. 4), but the swarm as a group does not change its position over time.

**Figure 5:**
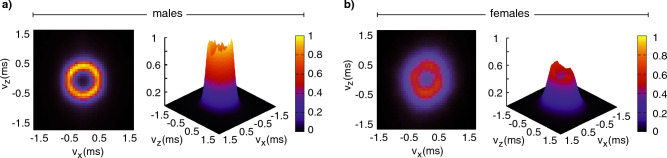
Group velocity probability distributions in the horizontal plane. (**a**) males. On the left, the top view of the probability distribution of the pairs (*v*_*x*_, *v*_*z*_) shown on the right. The radial symmetry displayed by the distribution confirms the absence of preferred directions of flight. The steep (*v*_*x*_, *v*_*z*_) distribution, corresponding to the thin yellow circle on the left plot, proves the high correlation of the two velocity components, which also shows that males tend to move at a constant speed in the horizontal plane, i.e. ${\text{v}}_{{\text{x}}}^{{2}} {\text{ + v}}_{{\text{z}}}^{{2}} {\text{ = constant}}$ (**b**) females. On the left, the top view of the probability distribution of the pairs (*v*_*x*_, *v*_*z*_) shown on the right. The distribution is much wider than in males. The well-defined thin circular ridge of the distribution found in males (panel a) is replaced by a thick blurred circle surrounded by a quite large region where *v*_*x*_ and *v*_*z*_ may lie, suggesting higher fluctuation of the speed in the horizontal plane.

In single-sex male swarms, we found a narrow distribution of *v*_*y*_, which reveals that motion mainly occurs on the *xz*-plane (parallel to the ground), confirming previously reported analysis of mosquito swarm dynamics in the field^27,41^ and, more generally, of lab swarms of other Diptera^42^. Flying with and against gravity imposes additional energy costs, which are minimized by limiting changes in flight altitude. On the other hand, the distributions of *v*_*x*_ and *v*_*z*_, with the two peaks at ± 0.5 m/s, give further information that there are no favoured directions of flight on the *xz*-plane, but all directions on the horizontal plane are explored with the same probability. Due to the planar nature of the motion, an equal probability of the direction of flight does not correspond to constant distributions of the two components of the velocity, which instead exhibit a characteristic trend, with two symmetric maxima at ± *v*, and a minimum at 0, see SI Section 1 where we show the double-peaked velocity distributions for a toy model of a particle moving with uniform circular motion in a plane. The absence of preferred directions of flight is also confirmed by the radial symmetry displayed by the probability distribution of the pairs (*v*_*x*_, *v*_*z*_), shown in Fig. 5.

**Figure 6:**
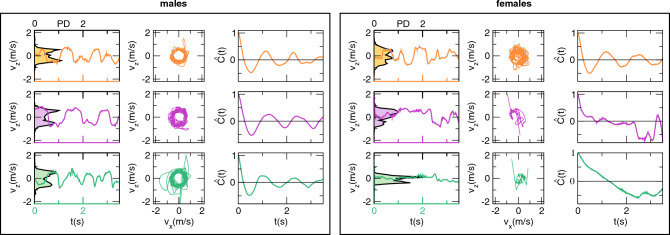
Individual velocity probability distributions and autocorrelation. Left box: males. Right box: females. First and fourth column: the evolution in time of the *z* component of the velocity for three males and three females. Time is indicated on the bottom, coloured horizontal axes of the graphs. The black lines filled with light colours represent the probability distribution (relative to the scale on the top, black horizontal axes). Second and fifth columns: individual mosquito velocity in the *v*_*x*_*v*_*z*_-plane*.* Third and sixth columns: individual velocity autocorrelation functions for three males and three females.

There are two potential interpretations of this data. One possibility is that individual mosquitoes perform zig-zag paths, each moving along a few different directions of flight during the relatively short recording time, and that it is the swarm as a whole that uniformly covers all the directions on the *xz*-plane. In this case, probability distributions of individual mosquitoes would not present the symmetric double peak that we found in group distributions. An alternative possibility is that each individual explores all the directions of flight by itself, performing consecutive ring-like movements. In the second scenario, individual mosquitoes would present probability distributions with the two peaks, which would only be reinforced by a larger statistical sample in the aggregated distributions. To discriminate between these two scenarios, we looked at the evolution in time of *v*_*z*_ and at probability distributions at the individual level (Fig. 6, *first column*), and found the same trend observed at the group level (Fig. 5). In Fig. 6 (*first column*), we show these quantities for three different mosquitoes, each displaying *v*_*z*_ with a periodic pattern in time and a double-peaked probability distribution characteristic of pseudo-circular planar motion. Plotting individual velocities on the *v*_*x*_*v*_*z*_-plane, we find that the two velocity components are highly correlated lying on a thin circle, Fig. 6 (*second column*), confirming the radial symmetry on the *v*_*x*_ *v*_*z*_-plane found at a group level (Fig. 5a). We also computed individual velocity autocorrelation functions (defined in detail in SI Section 2) that, by measuring the degree of alignment between velocity vectors of a single mosquito at different instants of time, represent a valid tool to detect repetitive patterns. These autocorrelations show periodic oscillations, which are similar in terms of amplitude and period for all individuals, see Fig. 6 (*third column*). This oscillating trend further highlights the ring-like or circular patterns exhibited by individual mosquitoes.

Females apparently display a different motion, with the distributions of the three components of the velocity wider than in males, Fig. 4 (*second row*). The double peak of the distributions of *v*_*x*_ and *v*_*z*_ is barely visible for female swarms, and the circle pattern, which characterized male distribution of the velocity on the *xz*-planes, is less evident suggesting a weaker correlation between the two velocity components, see Fig. 5. This apparent kinematic difference is evidence of the different approach to swarming of males and females. Males entering a swarm will join the group for a long time, generally longer than our recording time of 15.88 s. In contrast, only a small portion of the females stay in swarming formation for a long time. However, when they do participate in the swarm core, they perform ring-like movements very similar to those displayed by males. In contrast to the males, females quite frequently pass straight through the swarm (see VideoSI_1 and VideoSI_2), following high-speed (up to 2 m/s), straight-line trajectories.

The presence of these females, that only briefly join the group, affects the probability distributions of the velocities, making the velocity distributions wider, reducing the double peak of the PDs of *v*_*x*_ and *v*_*z*_, and reducing the correlation between *v*_*x*_ and *v*_*z*_, see Fig. 4. As in males, the distribution of (*v*_*x*_, *v*_*z*_) shows a radial symmetry, suggesting that also in females there are no favoured directions of motion. But, as shown in Fig. 5, the distribution is much wider than in males. The well-defined thin circular ridge of the distribution found in males (panel a) is replaced by a thick, blurred circle surrounded by quite a large region where *v*_*x*_ and *v*_*z*_ may lie, suggesting higher fluctuation of the speed in the horizontal plane.

Individual probability distributions and velocity autocorrelations, shown in Fig. 6, confirm the coexistence of two different populations of females. Some individuals, who are engaged in swarming in proximity to the ground marker, perform ring-like patterns similar to the ones we found in males (e.g. Figure 6, first female example—indicated in orange). In contrast, some other individuals (e.g. Figure 6, second and third female examples—indicated in purple and green), instead, seem to be uninterested in the marker and follow straight paths. The evolution in time of *v*_*z*_ (*column 4*) does not show a repetitive pattern, except for those mosquitoes participating in the swarm (orange line in Fig. 6). Even here, however, the periodic trend is barely visible. The two components of the velocity on the *v*_*x*_*v*_*z*_-plane (*column 5*) are correlated only for the females participating in the swarm (orange plot in Fig. 6). But the thin circle typically found in males *(*Fig. 6*, column 2*) is replaced by a larger and blurred circle, suggesting larger speed fluctuations in females than in males. We also found a large proportion of mosquitoes that do not show a correlation between *v*_*x*_ and *v*_*z*_ (purple and green plot in Fig. 6). Finally, the autocorrelation of the females participating in the swarm (e.g. the orange plot of Fig. 6, *column* 6), shows a periodic pattern, confirming that the movement of females participating in the swarms is similar to that of males, but we found many cases where correlation does not show any particular trend (e.g. the purple and green plots in the sixth *column* of Fig. 6), distinct from what is observed in males. This heterogeneity in female swarms, which we do not find in males, suggests a different exploration strategy adopted by individuals of the two sexes. Once released, males congregate in proximity to the ground marker and form large, stable and well-defined swarms. In contrast, the majority of females tend to explore a large area of the cage, generally flying solo and not being limited to the confined space around the marker.

### Mosquito encounters within swarms

**Figure 7:**
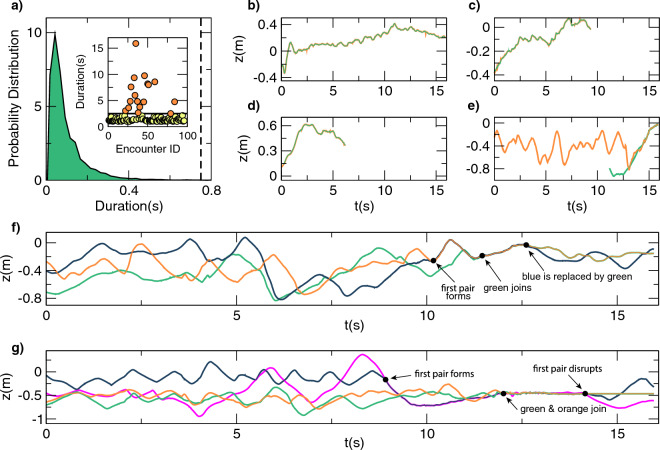
Male–female encounters. (**a**) From 19 reconstructed acquisitions of single-sex male swarms we extracted the probability distribution of the duration of male-male encounters, defined as the time spent by two mosquitoes at a mutual distance shorter than a threshold, chosen (for each swarm separately) as half the mean nearest neighbor distance, namely in the range between 5.9 cm and 12.6 cm (see Table SI1). Maximum duration of male-male encounters is 0.75 s, indicated with a vertical dashed line. In the inset: for 8 mixed-sex swarms, encounters lasting more than 0.75 s, which are unlikely to be found in single-sex male swarms, are labelled with a unique ID and their duration is shown. Of these encounters 80% (79 out of a total of 96), highlighted with yellow circles, last less than 2.5 s, the horizontal black line. The other 20%, highlighted with orange circles, last more than 2.5 s. They may correspond to male–female mating events. (**b**–**g**) the *z*-component of the trajectories of mosquitoes involved in some of the long-lasting encounters are shown as a function of time. Video footage of these encounters is available online. (**b**) E1: the longest encounter, lasting 15.88 s (**c** and **d**) E2 and E3: the two encounters last 9.3 s and 6.2 s, starting before the beginning of the acquisition and ending when mosquitoes exit the common field of view of the cameras. (**e**) E4: the encounter starts at the very end of the acquisition. The trajectory highlighted in green is presumably a female joining the swarm a few seconds after the acquisition started. (**f**) E5: three mosquitoes engaged in a mating competition. A first pair, orange and blue trajectories, is joined by a third mosquito (green trajectory), which is able to replace the blue individual in the established pair. (**g**) E6: four mosquitoes engaged in a mating competition. One pair, blue and magenta, is joined by two other mosquitoes, denoted by orange and green. The four briefly fly together. The competition ends with the orange-green pair landing on the ground, and the first formed pair disrupted, with the blue and magenta mosquitoes separately returning to the swarm.

Having defined some of the characteristics of single sex swarms we wanted to see whether we could document apparent mating encounters. Males (approx. 1500) were released into the swarming cage and allowed to acclimatize as described above. Once the daylight illumination was extinguished and males spontaneously began to swarm, virgin females were added in two batches of 250 at a time. Since, from the video footage we collect, there is no way to establish the gender of two mosquitoes potentially interacting in flight, we needed some objective and quantitative measure of encounters taking place in single sex swarms compared to mixed swarms. We therefore calculated the mean ‘distance to nearest neighbor’ for each of the 19 reconstructed swarms of only males (a distance between 5.9 cm and 12.6 cm depending on the swarm, see Table SI1). For each swarm we defined an ‘encounter’ as two mosquitoes flying at a mutual distance within half this value. We analyzed all the reconstructed acquisitions of single sex male swarms to establish the maximum duration of male-male ‘encounters’. As depicted in Fig. 7a, the vast majority of ‘encounters’ lasted less than 0.2 s while no encounters in male only swarms were found to last longer than 0.75 s. We therefore performed the same analysis on mixed swarms, and isolated those encounters lasting more than 0.75 s: events that do not occur within male-only swarms. We identified 96 encounters in mixed swarms that surpassed the 0.75 s duration threshold and each was given an identifying number. In Fig. 7a (inset) we plotted the duration of each of these 96 interactions against its identifying number. We observed that approximately 20% of these encounters (17 of the 96 events) have a duration greater than 2.5 s.

Since 11 of the 17 stable and long-lasting encounters belong to the same swarm (acquisition 20200928_ACQ6—generation 2) we focused on this acquisition, and investigated encounters by comparing images and 3D reconstructed trajectories. For 6 of these events (E), we present, in Fig. 7, the z-components of the trajectories involved and, in the videos (VideoSI_3, VideoSI_4, VideoSI_5, VideoSI_6, VideoSI_7, VideoSI_8) available online, their evolution in time. Three of these events (E1–E3) involve the trajectories of two mosquitoes flying in pair formation. E1 (Fig. 7b), the longest encounter we found, lasts for 15.88 s (all 2700 recorded frames of the acquisition). E2 and E3 (Fig. 7c,d) are shorter, lasting 9.3 s and 6.2 s respectively, namely from the beginning of the acquisition until, still flying in pair formation, they leave the common field of view of the cameras.

A fourth event, E4 (Fig. 7e), represents an encounter between one mosquito, who stays within the swarm from the very beginning of the acquisition, and a second that enters the common field of view of the cameras at 11.2 s. The encounter starts 3.4 s after this second mosquito, presumably a female, joins the swarm, and it lasts until the end of the recording.

The last two events (E5 and E6) document apparent attempts by third parties (presumably males) to interrupt established couples. E5 involves three mosquitoes, whose trajectories are highlighted in orange, green and blue in Fig. 7f and in the corresponding video online (VideoSI_7). The first pair, orange and blue, starts at 10.2 s. At 11.3 s, the green mosquito approaches the other two, and at 12.8 s, is able to replace the blue mosquito in the pair. Finally, in E6 (Fig. 7g), there are four mosquitoes (orange, green, blue and magenta). At 8.8 s, blue and magenta engage. They fly together until, at 12.1 s, the orange and the green mosquitoes, which were flying solo, approach the established pair. The four mosquitoes stay together for a short time before crash-landing on the ground. The original couple (blue and magenta), now separated, fly off, returning to the swarm, while the two presumptive interlopers remain on the ground. Together these data indicate that inter-mosquito encounters taking place within mixed swarms have a unique, although variable, duration, i.e. longer-lasting than any seen in single sex swarms. The data also document competitive behaviors consistent with the idea of ‘scramble competition’ between males, believed to characterize mosquito swarms^5,43^.

## Discussion

Swarming behavior is central to the reproductive capacity of anopheline mosquitoes, thereby controlling the size of the vector population and its capacity to spread malaria-causing parasites. For the same reason swarming is also pivotal to the sexual transfer of transgenes or bacteria aimed at reducing parasite transmission that underpin long-awaited gene drive and endosymbiont approaches to vector control^9–13^. We set out to shed new light on the individual and group dynamics that define anopheline swarming and mating behavior by studying lab-based swarms using 3D-video tracking. Specifically, we aimed to characterize individual and group motion in single-sex swarms that could be used as a benchmark for future comparison with genetically-modified mosquitoes for example, and at the same time, to quantify differences in swarming behavior of males and females reported in^22^ and to document mating-related encounters in mixed-sex swarms.

The approach we describe allowed measurement of the individual and population level dynamics of mosquito swarms containing more than 200 participants with a frame rate of 170fps. In male swarms 73.3% of all tracks lasted more than 14 s of the acquisition. Previous 3D tracking of field-based swarms produced average track lengths of 0.84 s (21 frames) due partly to the restricted frame rate (25fps), and consequently lower resolution, of the moving mosquitoes^39^. Moreover, some of the swarms selected for analysis in those studies contained less than 50 individuals which facilitates tracking but may also have impacted statistical validity. Thus, the results reported here describe mosquito swarming behavior in unprecedented detail.

One a priori methodological concern was whether the illumination of the swarming arena with infrared light, necessary to visualize the mosquitoes, might interfere with swarming behavior. Previous work suggests mosquitoes may be sensitive to light in the infrared range^44^, albeit the wavelength of infrared light used here (850 nm) would be at the limit of that detectable by mosquitoes. However, importantly, the timing, duration and gross structure of the swarms we observed here, in the presence of infrared light, were indistinguishable from those we have described previously in a very similar setting, in its absence^40^. This is consistent with published data documenting apparently authentic host-seeking behaviors in the presence of infrared light^45,46^. Moreover, as discussed below, the swarming behaviors we documented here are consistent with those documented in naturally-illuminated field^39,47^ and semi-field settings^22^.

Because with 3D-tracking we reconstruct the individual trajectories of all the participants in a swarm, it is possible to compute their velocities in three dimensions for the entire acquisition. We can then characterize mosquito motion by analysing the velocity probability distributions in the three directions of motion as well as the autocorrelation functions. This allowed us to investigate the movement of individuals within the swarm, revealing its simplicity; it is in general restricted to the horizontal plane and takes the form of repetitive, pseudo-periodic movements (see Figs. 3, 4, 5 and 6). Moreover, most clearly in the case of males (Fig. 6), population level analyses reveal that this pattern of movement is essentially common to all members of the swarm.

Given the reported impact of lab-adaptation on some mating behaviors^48^, the relevance of our experimental setup in benchmarking mosquito swarming depends also on the consistency of our results with those reported in the field. Several elements of male motion within lab-adapted swarms captured here are in agreement with data measured in the field. The average velocity of males that we measure across different swarms and different generations is comparable with field measurements^39^, the dominance of male movement in the horizontal plane has already been quantitatively proved^27,41^, while the correlation between the number of males released in to the cage and the number participating in the swarm, shown in Fig. 1g, has also been observed in a semi-field setting^22^. Finally, the cylindrical or barrel-like shape of male swarms (Fig. 2) and the ‘figure of eight’ form of male motion, similar to the repetitive ring-like movements we found, were observed in the field at a qualitative level in^47^, namely looking by eye at different swarms.

Our study also provides documentary evidence of apparent swarming-like behavior in female-only groups. These ‘swarms’ consist of a tight swarming ‘core’, above which is a zone characterized by much more chaotic motion. This dichotomy in the nature of female swarming behavior limits the utility of describing their group level behavior in a single statistic. For example, while the average speed of males could usefully be measured (0.59 ms^−1^), as shown in Fig. 4, the same statistic for females, being a composite of two distinct populations, has little value. In contrast, the reconstructed trajectories and population-level statistical analysis of those trajectories (Fig. 4) provide a much clearer rendering of the complexity of female ‘swarms’. Indeed, while the sharply demarcated probability distribution of male velocities (Figs. 5 and 6) is ‘blurred’ in the case of females by a large number of rapid and chaotic movements, it is still possible to discern that many females, presumably those residing within the swarm ‘core’, in fact exhibit pseudo-periodic motions akin to those of males. As with the motions of males described above, we believe these quantitative descriptions of female swarming behavior to be novel insights and yet, these lab-based female ‘swarms’ share several features with female-only aggregations observed in a semi-field setting^22^. These include lower numbers of participants relative to male swarms; more fleeting associations with the ‘swarm’ core located in proximity of a ground marker; features reminiscent of previously reported ‘offering flights’^49^.

Beyond individual and group swarming dynamics we were also able to document multiple apparent mating-like encounters. While, in the current set up, we are unable to distinguish males from females, we have some confidence that the encounters observed in mixed swarms were probably between males and females: in single sex swarms we failed to observe a single incidence of a pair of mosquitoes interacting (remaining separated by less than half the mean distance to nearest neighbor) for more than 0.75 s. Instead, in mixed swarms we observed a total of 96 encounters that surpassed this threshold which included 17 examples where the encounter lasted for more than 2.5 s. Could these encounters of different durations have some functional significance? Specifically, since the longest-lived encounters we detected (approx. 10 s) approach estimates of the duration of copulation in this species^17^, it is tempting to speculate that these encounters may represent successful mating events.

In addition to these long-lived encounters (≥ 2.5 s) we detected many more (79/96) that were more fleeting, lasting for between 0.75 and 2.5 s; probably insufficient time to enable mating to take place yet significantly longer lasting than any detected in single sex swarms and thus likely to represent encounters between males and females. There are several possible explanations for these ‘brief encounters’. One is that they are essentially long-lasting encounters, artificially curtailed by beginning or ending outside the 15.88 s acquisition period. An alternative interpretation is that at least some of these meetings may represent male–female encounters that fall short of copulation. Implicit in such encounters, should they be verified in future studies, is a degree of mate choice, a possibility also supported by lab-based observations such as kicking behaviors in tethered females and avoidance behaviors in free-flying females^17^, as well as ‘courtship-like’ signals such as the repeated close contacts that precede successful mating-couple formation in field swarms^39^.

Our results also provide direct documentary evidence of apparent male-male mating competition. While such behaviors are predicted from evolutionary models of reproduction in swarms^28^ and have been reported in observational studies of wild anopheline swarms^50^ (and references therein), to our knowledge this is the first recording of such events in a natural swarm setting. Male competition is believed to be a crucial determinant of male mating success^43^, a variable that is central to predictions, for example, of how a genetic modification in a gene-drive mosquito line might spread through a population. While the limited number of examples in the current dataset preclude objective analysis, in future these findings may offer a means of quantifying mating fitness in different male mosquitoes.

In conclusion, with this work we take the first steps towards the characterization of lab-based swarms of *Anopheles gambiae*, providing new insight into the complexity of mosquito motion. The agreement of our results with previously reported quantitative analysis and observational studies of field swarms confirms that, despite the controlled conditions of the laboratory, our experimental setup reproduces pseudo-natural swarming behaviors. On this basis, the characterization of mosquito kinematics presented here represents a useful benchmark to validate swarming models and to test how novel vector control measures may impact swarming behaviour.

## Materials and methods

### Mosquito husbandry

Mosquitoes were contained in a containment level 2 facility at Department of Medicine and Surgery, Perugia, Italy, authorization N. PG/IC/Imp2/13/001-Rev2 from Ministry of Health.

**Figure 8:**
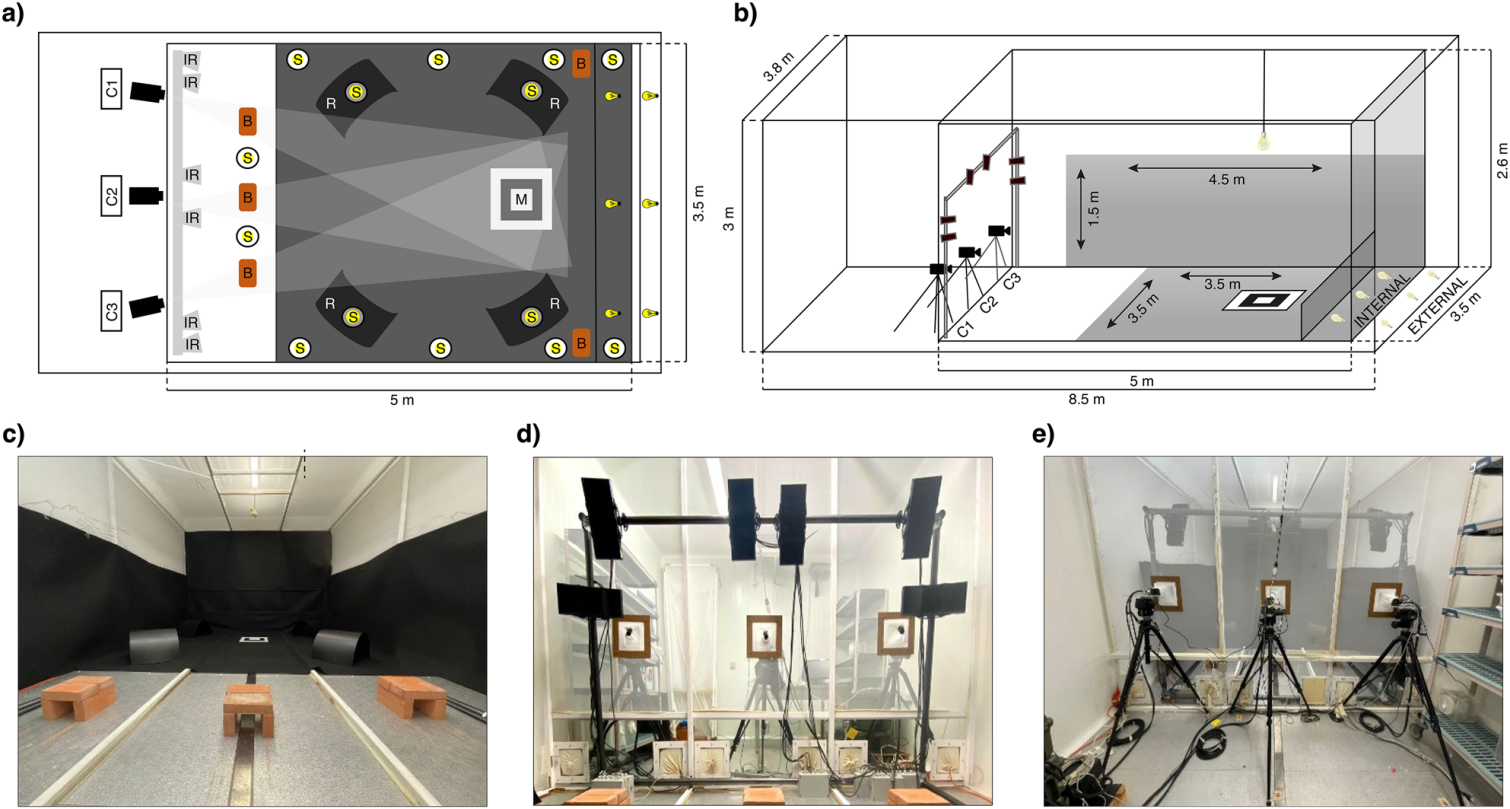
Schematic representation and pictures of semi-field insectary chamber. (**a**) Schematic view of the semi-field setup. The swarming stimuli, feeding and resting stations comprise: the two sunsets; visual ground marker (M, dimensions: internal square 20 cm × 20 cm, central square 40 cm × 40 cm, external square 55 cm × 55 cm); sugar sources (S); mosquito resting shelters (R) and terracotta brick resting shelters (B). The infrared lights (IR) are placed inside the large cage (also depicted in panel d). Cameras (C1, C2, C3), located outside the cage, are directed towards the visual ground marker. (**b**) 3D rendering of the swarming chamber. The image shows the dimensions of the chamber containing the large cage in which swarming takes place; dimensions and location of the infrared-absorbing black cloth wall coverings; position of the warm light above the marker; internal and external sunsets; the six infrared LED lamps supported by an iron scaffold; the 3 video cameras (C1, C2, C3); contrasting ground marker with the three white-black-white plastic sheets. (**c**) A picture of the setup from within the cage, showing mosquito resting shelters. In the foreground are the terracotta brick resting shelters, in the background two of the black resting shelters and the swarming ground marker. The interior walls of the cage are covered with black cloth to produce a uniform background in the images. (**d**) A picture of the six IR lamps from inside the cage, with cameras in the background, outside the cage. The three squares on the net are the holes where camera lenses are positioned. (**e**) A picture of the three cameras from outside the cage.

*Anopheles gambiae (An. gambiae)* G3 strain (MRA-112) are reared using the MR4 protocol^51^; 28 ± 1 °C and 75 ± 10% relative humidity under a 12/12-h light/dark cycle with ad libitum access to 10% w/v glucose solution supplemented with 0.1% w/v methylparaben. Females 3–5 days old, receive a meal of bovine blood (source: Allevamento Blood di Ricci Chiara, Teramo, Italy) on a Hemotek PS5 membrane feeder system. Collected eggs are bleached to prevent possible infection, allowed to hatch, then larvae transferred at a density of 215 ca. to plastic trays with 350 ml of deionized water. The larvae are maintained on a tuna/liver/vitamin slurry (2:2:1 respectively)^52^. During the aquatic phase of mosquito development juvenile males and females can be distinguished at the pupal stage by microscopic examination of terminalia. This allows separation of males and females, before adulthood is reached, and thus the generation of single-sex colonies of ‘virgin’ mosquitoes. For each experiment, between 800 and 1800 2–3-day-old virgin males or females derived from a single mosquito generation (i.e. the product of a single egg batch) were released from ‘Bug-Dorm’ type cages (35 cm × 35 cm × 35 cm) into the swarming enclosure (5 m × 3.5 m × 2.6 m, shown in Fig. 8) approximately 1 h before the onset of the artificial sunset.

### System set up

Building on our previous studies of lab-based swarms^40^ and experience of field swarms in other insects^36^ we record mosquito swarming behavior using three synchronized but independently positioned high-speed video cameras. We have previously described robust and repeatable swarming behavior using a large, enclosed room containing an artificial horizon and a black and white ground marker above which swarming activity was focused^40^. To reduce the risk that the size of the room should affect swarming behavior, we expanded three-fold, the volume described previously as well as the dimensions of the ground marker whilst retaining the artificial horizon (Fig. 8a) and adding both sheltering locations and sugar feeding sources (Fig. 8b). To provide sufficient light conditions without disturbing mosquitoes^46^ (see ‘Illumination’ paragraph below), we illuminate the swarming area with six infrared lights. By individually calibrating each camera (described below), and the application of tracking algorithms described previously^37^, combining images from each of the three video cameras, we were able to reconstruct position and compute velocity in 3D space of each individual mosquito in a swarm while resolving the frequent occlusions that occur in such a feature-dense environment. The algorithm enabled the successful tracking of 73.3% of the participating mosquitoes, generating up to 15.88 s of mostly unbroken footage.

### Large cage design

Data collection took place in the Insectary Field 1 (measuring 8.5 m × 3.8 m × 3 m, L × W × H) at the Department of Medicine and Surgery of the University of Perugia, which contains one large cage of 5.0 m × 3.5 m × 2.6 m (Fig. 8a). It consists of white-painted wooden frames with walls and ceiling made of polyester mesh as previously described^40^. Since swarms occur naturally at dusk, the experiments are illuminated using infrared light (IR), allowing the three synchronized cameras to capture mosquito motion even in low light conditions. The floor, the back and the two side walls of the cage are covered with black cloth providing a dark, non-reflective background, against which, the mosquitoes appear as white dots when illuminated directly by the IR (Fig. 8a). Six 850 nm IR LED lamps (RAYMAX 300) are supported by an iron scaffold located inside the cage, immediately adjacent to the three cameras (Fig. 8a). The lamps are oriented towards the swarming area such that the camera field of view and the area of optimal illumination are coincident with the ground marker over which the mosquitoes congregate and swarm in a highly repeatable manner.

### Mosquito feeding

As depicted in Fig. 8b, the large cage contains four dome-shaped polypropylene sheets (R), located at the four corners; five terracotta shelters (B) at the front (camera side) of the cage and two in the corners adjacent to the sunset, which are kept humid to serve as damp resting sites, where mosquitoes spend the majority of the time. Sugar feeding is energetically critical for mosquitoes, since swarming activity has a significant energy cost^53^. To ensure adequate energy reserves in swarming mosquitoes, fourteen sugar sources consisting of Petri dishes filled with cotton wool soaked in sugar solution (10% glucose solution, 0.1% methylparaben as preservative) are placed at various points of the cage. In addition, approximately 4 ml of acacia honey is added, as an aromatic attractant, on white absorbent paper placed on top of the sugar-soaked cotton wool.

### Reproducing natural swarming stimuli

Several visual stimuli are required for swarming to occur under artificial conditions^40,54^. As shown in Fig. 8b, the swarming stimuli in the large cage comprise: (i) a contrasting ground marker (M) containing three concentrically arranged square plastic sheets white-black-white (55 cm × 55 cm, 40 cm × 40 cm and 20 cm × 20 cm, respectively) in the back half of the cage, at approximately 1.1 m from the back wall; (ii) two series of lights: three 2.700 K 8 W lights located on the floor outside the cage (External Sunset, ES) shining upward onto the wall of the insectary chamber, and (iii) three 2.700 K 9W LED lights placed on the floor at the back of the cage to simulate twilight (Internal Sunset, (IS), which are protected by plastic sheets (55 cm high) covered with non-reflecting black cloth to prevent the light from shining directly into the cage. The staggered switch-off of the external and internal sunsets produces a softer, more even light spread, more faithfully mimicking field conditions. A 2700 K 2.8 W warm LED light is placed immediately above the swarm marker to make it more visible and attractive.

### Video recording system

Data is collected using a stereometric camera system consisting of three high speed synchronized cameras (IDT-MotionScope M5 shooting at 170fps) equipped with Schneider Xenoplan 28 mm f/2.0 optics. Each camera is placed on a plexiglass shelf, supported by a metal tripod (Manfrotto Pro Digital Tripod 475B), allowing the correct orientation of the cameras towards the swarming area. The three cameras are located outside of the large cage at a distance of 3.5–3.9 m from the ground marker. The three lenses project through holes in the net and focus on the ground marker. See Fig. 8b.

### Illumination

Illumination is one of the main criticalities for 3D tracking laboratory experiments. Particular care has to be taken to recreate light conditions that reproduce a suitable environment for swarming and, at the same time, guarantee high-quality images at a high camera frame rate (170fps in our set-up). These two aspects are in conflict with each other: swarms generally form at dusk, hence we need to work in low light conditions, which makes image contrast poor unless a long exposure time is chosen. But exposure time is linked to the frame rate, being limited by the time between two consecutive frames. Shooting at 170fps exposure time cannot exceed 0.006 s, which is too short to make mosquitoes properly visible on the images. Therefore, in order to detect mosquitoes we need to add a source of light, ideally one that may be captured by the cameras but is not visible to the mosquitoes, which otherwise may not swarm. The seminal work by Gibson^44^, addresses the question of whether mosquitoes are sensitive to light at different wavelengths, and concludes by stating that laboratory experiments should be performed with infrared at a wavelength > 900 nm. We investigated the quantum efficiency of our cameras (IDT-M5) at that specific wavelength, and found that it would have been too low. We therefore opted for a compromise, choosing infrared lights at 850 nm as in^45^, thus close enough to the threshold indicated by Gibson and with an acceptable quantum efficiency for our cameras.

### 3D reconstruction

The cameras record the same swarming event from three different points of view enabling reconstruction of the 3D position of each object in the common field of view of the cameras. To ensure high accuracy in the reconstructed position, the baseline of the camera system, i.e. the relative distance between the two lateral cameras, is set to 2.5 m. With these parameters, the 3D resolution of the system is equal to 1 mm and the typical reconstruction error is below 0.2%^55^.

### System calibration

When used as quantitative tools, cameras must be calibrated to guarantee that the reconstructed scene matches the metric properties of the real world, such that lengths and angles can be measured upon reconstruction and have their veridical values. A first calibration routine has to be performed on each camera separately, to define the camera’s internal parameters, namely the focal length, the position of the optical centre and the distortion coefficient^56^. In order to minimize reconstruction error with stereo camera systems, it is also essential to define the external parameters, i.e. relative position and orientation of the cameras. We obtain these external parameters with a post-calibration procedure^36,55^, already successfully used to collect field data on midge swarms.

### 3D Tracking

Once the images are collected, they are analyzed through the post-processing software GReTA^37^, which is a global and recursive tracking algorithm that guarantees high performance in terms of trajectory length and produces a negligible rate of identity switches (mistaking one mosquito for another).

### Counting mosquitoes participating in a swarm

We counted the number *N* of mosquitoes participating in each acquisition, detecting mosquitoes in representative still images via the segmentation software described in^37^. We compared *N* of different acquisitions belonging to the same generation, to express, for each generation a number *Nmax* that quantifies how many mosquitoes reacted to our swarming stimuli, even for a short time. Specifically, since different acquisitions in the same generation represent the same swarm over time, we defined *Nmax* as the peak swarm participation, that is the maximum number of participating mosquitoes in the set of acquisitions belonging to a given generation.

## Supplementary Information

Supplementary Information 1.

Supplementary Video 1.

Supplementary Video 2.

Supplementary Video 3.

Supplementary Video 4.

Supplementary Video 5.

Supplementary Video 6.

Supplementary Video 7.

Supplementary Video 8.

## Data Availability

The data that support the plots within this paper and other findings of this study are available from the corresponding authors upon request.
