# Supplementary material for: Characterization of lab-based swarms of Anopheles gambiae mosquitoes using 3D-video tracking
Source: Sci Rep. 2023 May 30;13:8745. doi: 10.1038/s41598-023-34842-0 (PMC10229557; doi:10.1038/s41598-023-34842-0)
Supplement: Supplementary file 1 — Supplementary Information 1. [file 41598_2023_34842_MOESM1_ESM.pdf]

Supplementary Information
Preliminary characterization of lab-based swarms of Anopheles gambiae mosquitoes using 3D-video tracking

Andrea Cavagna[1,2,3], Irene Giardina[1,2,3], Michela Anna Gucciardino[4,5], Gloria Iacomelli[4], Max Lombardi[4], Stefania Melillo[1,2], Giulia Monacchia[4], Leonardo Parisi[1,2], Matthew J. Peirce[4], and Roberta Spaccapelo[4,5]

[1]CNR–ISC (National Research Council - Institute for Complex Systems), Rome, Italy
[2]Physics Department University Sapienza, Rome, Italy
[3]INFN, Unità di Roma 1, Rome, Italy
[4]Dipartimento di Medicina e Chirurgia, Centro Universitario di Ricerca sulla Genomica Funzionale (C.U.R.Ge.F), University of Perugia, Perugia, Italy
[5]Centro Universitario di Ricerca sulla Genomica Funzionale (C.U.R.Ge.F), University of Perugia, Perugia, Italy

1 A toy model: uniform circular motion.

In Section 2.2 of the main article, we provide a characterization of male mosquitoes' motion based on the analysis of the velocity probability distributions, and of the autocorrelation functions shown in Fig.4, Fig.5 and Fig.6: males essentially fly at a fixed altitude, performing consecutive, ring-like patterns in horizontal planes parallel to the ground. The narrow probability distribution of the y-component of the velocity, the one parallel to the direction of gravity shown in Fig.4a, indicates little deviation of v_y from 0 or, equivalently, only slight variation of the direction of motion with or against the gravity, hence an almost constant flight altitude. On the other hand, the double-peaked distributions of v_x and v_z, shown at a group level in Fig.4b and Fig.4c and at an individual level in Fig.6 (third column), suggest a pseudo-circular motion in the horizontal plane. This correspondence between double peaked distribution and circular motion might not be intuitive to a general audience. Therefore, to aid understanding of the results presented in Section 2.2, we introduce here a simple but instructive toy model, a single particle following a planar uniform circular motion. By computing probability distributions of the two components of the velocity in this toy case, we will clarify the agreement of the two peaks in the velocity distribution with the ring-like patterns of mosquito motion we observed.

Our toy model is a single particle moving along a circular path, of radius r, at a constant angular speed, ω, in the counterclockwise direction. The particle motion is periodic with period $T = 2\pi/\omega$. For consistency with the results shown in the main article, we assume that the particle moves on the xz-plane, see Fig.SI1. We measure its angular position, α, as the angle between the x-axis and the radius passing through the particle. We assume that at time $t = 0$, the particle lies on the x-axis, so that $\alpha(t) = \omega t$, which is linear in time, with α going from $0 rad$ to $2\pi rad$ in the time interval $[0, T]$, see Fig.SI1a.

The $2D$ position of the particle, $\vec{x}(t) \equiv (x(t), z(t))$ is described by the following equations of motion:

$$\vec{x}(t) = \begin{cases} x(t) = r\cos(\omega t) \\ z(t) = r\sin(\omega t) \end{cases} \tag{1}$$

The two components, $x(t)$ and $z(t)$ highlighted in blue and red respectively in Fig.SI1d, are sinusoidal in time, with an amplitude equal to r and a period equal to T.
The derivatives in time of Eq.1 give the velocity vector, $\vec{v}(t) \equiv (v_x(t), v_z(t))$, of the particle, which is then defined as:

$$\vec{v}(t) = \begin{cases} v_x(t) = -r\omega\sin(\omega t) \\ v_z(t) = r\omega\cos(\omega t) \end{cases} \tag{2}$$

As noted for the particle's position, the components of its velocity are also of a sinusoidal form with period T, but with amplitude ωr, see Fig.SI1b and Fig.SI1e. On the $v_x v_z$-plane the velocity vector, moves in time along the circle of radius $v = \omega r$. The two yellow circles in Fig.SI1a and Fig.SI1b represent respectively position and velocity of the particle at time $t = 0$: the

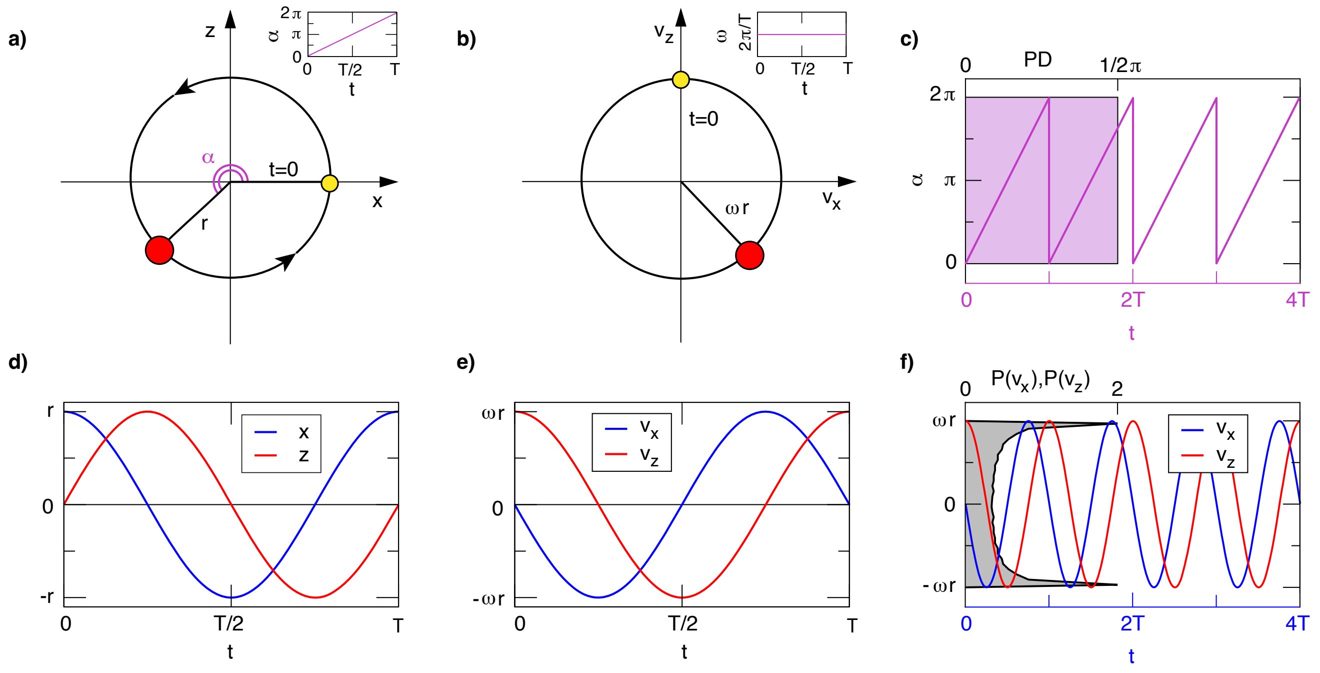

Fig.SI 1. Uniform circular motion. a) A single particle moves, in the xz-plane, along a circular path of radius r at a constant angular velocity ω, corresponding to a revolution period $T = 2\pi/\omega$. The angular position, α, is measured as the angle between the x-axis and the radius passing through the particle, in the counterclockwise direction. At time $t = 0$ the particle lies on the x-axis, highlighted with a yellow circle. The red circle represents the position of the particle at a generic time t. **In the inset:** $alhpa$ is linear in time, going from $0rad$ to $2\pi rad$ during the time period T.**b)** In the v_xv_z-plane, the velocity of the particle lies on a circle. The yellow circle represents the velocity of the particle at time $t = 0$, the red circle its velocity at a generic time t. **In the inset:** the angular velocity of the particle is constant. **c)** The purple line represents the angle α as a function of time, relative to the purple scale on the bottom horizontal axis. The saw tooth shape is due to the equivalence between α and $\alpha + 2\pi$. The black line filled in light purple represents the probability distribution of α, relative to the scale on the black horizontal axis at the top. The angle is uniformly distributed, with the probability distribution constant and equal to $1/2\pi$. **d)** The two components of the position of the particle, as a function of time. The blue line represents the x coordinate, the red line the z coordinate. They are both sinusoidal functions. **e)** The two components of the velocity of the particle, as a function of time. The blue line represents the x coordinate, the red line the z coordinate. As noted for the position, both components of the velocity also have a sinusoidal trend in time. **f)** The blue and red lines represent the two velocity components as a function of time, relative to the scale on the bottom horizontal axis. The black line filled in grey represents the probability distribution of the two components of the velocity, which are not constant because of the flat derivative of the sinusoidal function in proximity of ± 1.

position of the particle is $x = 1$ and $z = 0$, hence its velocity is such that $v_x = 0$ and $v_z = 1$. In the same way, when the angular position of the particle is equal to α, its velocity vector, on the v_xv_z-plane corresponds to the vector at an angle $\alpha + \pi/2$.

1.1 Velocity probability distribution

In our toy model, the particle moves at a constant angular speed ω, therefore its angular position α is linear in time, $\alpha(t) = \omega t$, over a period of time T, and displays the periodic saw tooth trend shown with the pink line in Fig.SI1c, over an interval of time longer than T. Its probability distribution, depicted as the light pink solid graph in Fig.SI1c, is constant and equal to $1/2\pi$. On the other hand, the two components of the velocity, v_x and v_z shown with the red and blue lines respectively in Fig.SI1e, follow a sinusoidal trend in time. Their probability distributions, represented in Fig.SI1f as a solid light grey graph, are equal: they both present a minimum at 0 and two symmetric maxima at $\pm r\omega$. Therefore, the uniform probability distribution of the angle is not reflected in a uniform distribution of the two velocity components. To better understand how this discrepancy arises, in the following we will first derive the analytic form of the distribution of the velocity components, and then give an intuitive interpretation. Finally, we will show that this distribution is compatible with the mosquitoes' velocity probability distributions presented in the main text.

1.1.1 Analytic expression

From an analytic point of view, the probability distribution of the angle α, $p_\alpha(\alpha)$ shown in Fig.SI2a, is defined as:

$$p_\alpha(\alpha) = \begin{cases} 1/2\pi & \alpha \in [0,2\pi] \\ 0 & \text{otherwise} \end{cases} \tag{3}$$

which is constant and equal to $1/2\pi$ in the interval $[0,2\pi]$.

The two components of the velocity, defined in eq.2, may be expressed in terms of α as:

$$\vec{v}(\alpha) = \begin{cases} v_x(\alpha) = -r\omega\sin(\alpha) \\ v_z(\alpha) = r\omega\cos(\alpha) \end{cases} \tag{4}$$

For the sake of simplicity in the notation, we will first compute the probability distribution of the variable $y = \cos(\alpha)$, and only at the end will we generalize the result to give an explicit expression for the two probability distributions of v_x and v_z, which will differ from $p_y(y)$ only by a scale factor. The probability distribution of the variable y, $p_y(y)$, and the probability distribution of α, $p_\alpha(\alpha)$ are related by the following equation:

$$p_y(y)dy = 2p_\alpha(\alpha)d\alpha \tag{5}$$

where $d\alpha$ and dy represent the differential of the two variables α and y respectively, and the factor 2 is needed to take into account that each value of the variable y corresponds to two distinct value of α, see Fig.SI2.

Eq.5 is equivalent to:

$$p_y(y) = 2p_\alpha(\alpha)\left|\frac{d\alpha}{dy}\right| = 2p_\alpha(\alpha)\left|\frac{dy}{d\alpha}\right|^{-1} \tag{6}$$

where $d\alpha/dy$ is the derivative of the variable α with respect to y, $dy/d\alpha$ is the derivative of y with respect to α, $|\cdot|$ denotes the absolute value, and the superscript -1 indicates the inverse. So that $|dy/d\alpha|^{-1} = |1/(dy/d\alpha)|$.

The derivative of $y = \cos(\alpha)$ with respect to the angle α, is given by:

$$\frac{dy}{d\alpha} = -\sin\alpha = -\sqrt{1-\cos^2\alpha} = -\frac{1}{\sqrt{1-y^2}} \tag{7}$$

Substituting eq.7 in eq.6, we obtain the analytic expression for $p_y(y)$

$$p_y(y) = \begin{cases} \dfrac{1}{\pi\sqrt{1-y^2}} & y \in (-1,1) \\ 0 & \text{otherwise} \end{cases} \tag{8}$$

$p_y(y)$ is the distribution plotted in Fig.SI2. It has a minimum at 0, where it is equal to $1/\pi$, and it grows symmetrically when y goes to 1 or -1.

Note that we find eq.8, also when considering the variable $y = \sin(\alpha)$, noting that in this case $dy/d\alpha$ is equal to $\cos\alpha$.

Therefore v_x and v_z have the same probability distribution that differs from $p_y(y)$ in eq.8 only by the overall scale factor $r\omega$:

$$p_{v_x}(v_x) = \begin{cases} \dfrac{1}{r\omega}\dfrac{1}{\pi\sqrt{1-v_x^2}} & v_x \in (-r\omega, r\omega) \\ 0 & \text{otherwise} \end{cases} \quad \text{and} \quad p_{v_z}(v_z) = \begin{cases} \dfrac{1}{r\omega}\dfrac{1}{\pi\sqrt{1-v_z^2}} & v_z \in (-r\omega, r\omega) \\ 0 & \text{otherwise} \end{cases} \tag{9}$$

1.1.2 Intuitive interpretation

The analytic expression for the variable $y = \cos\alpha$ in eq.8 clearly shows that, a uniform distribution of the angle does not correspond to a uniform distribution of sine and cosine. Here we want to give an intuitive interpretation of this result. We will refer to the cosine function, but the same interpretation holds for the sine function.

Why is $p_y(y)$ not constant? The cosine, as a function of the angle α, has a flat derivative when its value is close to ± 1 ($\alpha = 0,\pi$), while it has a derivative close to 1 when its value is close to 0 ($\alpha \sim \pm\pi/2$). Therefore, in proximity of $\alpha = \pi/2$, the cosine function is almost linear, and uniformly distributed values of the angle will remain almost uniform: an interval of size $\Delta\alpha$ in the angle α will correspond to an interval $\Delta y_{\pi/2}$ in the y variable that is almost equal to $\Delta\alpha$, see Fig. SI2. In contrast, when α is close to 0, the cosine function is not linear because its derivative is almost 0, and the same interval of size $\Delta\alpha$ is squeezed into a range, Δy_0, that is much smaller than $\Delta\alpha$, hence the density at Δy_0 is higher than at $\Delta y_{\pi/2}$. This higher density corresponds to a higher probability of $\cos(\alpha)$ in proximity of ± 1.

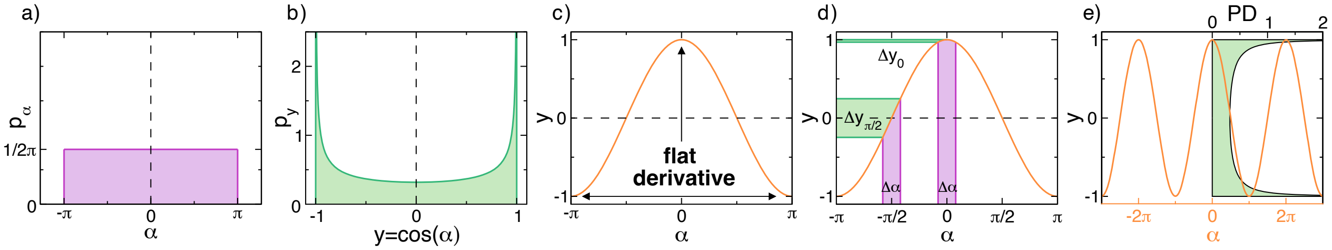

Fig.SI 2. Toy model - Probability distribution. a) the constant probability distribution of the angle α, defined in the interval $[-\pi,\pi]$. **b)** the probability distribution of $y=\cos(\alpha)$ is not constant, even when α is uniformly distributed. **c)** y has flat derivative when it is close to ± 1 ($\alpha = 0, \pm\pi$). The flat derivative implies a low sensitivity of y to a change in α. **d)** y as a function of α is linear when α is close to $\pm\pi/2$. An interval of size $\Delta\alpha$, centered on $-\pi/2$ is transformed, through the cosine function, to the interval $\Delta y_{\pi/2}$, which is almost equal in size to $\Delta\alpha$. Because of the flat derivative of the cosine when $\alpha = 0$, the same interval of size $\Delta\alpha$ centered on 0 is squeezed into the interval Δy_0. Δy_0 has a higher density than $\Delta y_{\pi/2}$. **e)** In orange: the variable y as a function of α (corresponding to the bottom orange horizontal axis). In black filled in light green: the probability distribution of y (corresponding to the top black horizontal axis). The regions where the derivative of y is flat correspond to regions where the density, and hence the probability, is high.

1.1.3 Consistency with real data

In Fig.4 of the main text, we plot the probability distribution of the three components of the velocity at a group level, obtained by aggregating mosquito velocities from 19 single-sex male swarms. In the x and z directions the probability distributions display a particular shape, with a minimum at $0m/s$ and a symmetric double peak at $\pm 0.5m/s$. We find the same double peak distribution in Fig.6, where we show the evolution in time of the z component of the velocity and the corresponding probability distributions for three different males. This trend in the probability distribution is consistent, although affected by noise, with the velocity distribution found in the toy model above, see Fig.SI1e. Experimental data are naturally affected by noise that partly comes from unavoidable $3D$ reconstruction inaccuracies, but which is mostly biological. Mosquitoes do not perform perfectly circular movements, they do not move at a perfectly constant speed, and they alternate circular movement and straight paths. Moreover, mosquitoes display fluctuations in velocity that are not present in our toy model, where the particle moves at a constant angular speed. All these sources of noise combine, through complicated mechanisms, to transform the shape of the velocity probability distributions, from the one displayed by the toy model to that observed in real swarms. The two sharp edges at the sides of the toy model distribution are replaced by the two distinct peaks in real swarms, and its rigid range of velocity, which cannot exceed $\pm r\omega$, is replaced by larger range due to mosquito fluctuations of the velocity components. Despite these differences, the toy model is consistent with the data, whose double peak distribution is therefore the effect of the mosquitoes' pseudo-circular motion.

2 Velocity autocorrelation function

In Fig.6 of the main text, we plot autocorrelation functions of three individual mosquitoes, and use their periodicity to confirm that mosquitoes' motion is characterized by ring-like patterns on the xz-plane. Velocity autocorrelation gives a measure of how much the direction of motion of a particle changes in time, and is a convenient tool for finding periodic and repetitive patterns in the particle path.

In order to determine the velocity correlation function, we start by computing $c(t_0,t)$ defined as:

$$c(t_0,t) = \vec{v}(t_0)\cdot\vec{v}(t_0+t) \qquad (10)$$

where $\cdot$ represents the scalar product.

$c(t_0,t)$ is equal to v^2 when $\vec{v}(t_0)$ and $\vec{v}(t_0+t)$ are perfectly parallel, equal to 0 when the two vectors are orthogonal, and equal to $-v^2$ when they are anti-parallel, see Fig.SI3.

The autocorrelation at time t, $C(t)$, is given by the average over t_0 of the quantities $c(t_0,t)$:

$$C(t) = < c(t_0,t) >_{t_0} \qquad (11)$$

so that $C(t_0) = v^2$. In order to compare autocorrelation functions, of objects moving at different speed, we normalized the correlation function by its value in $t=0$: $\hat{C}(t) = C(t)/C(0)$ in such a way that $\hat{C}(0) = 1$.

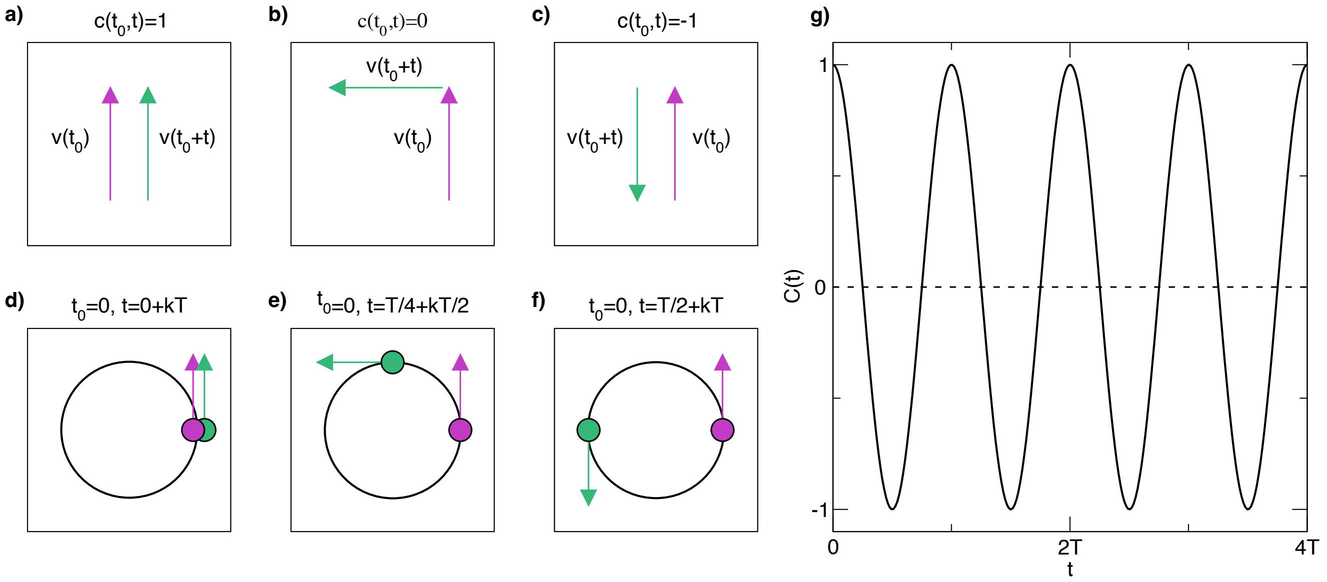

Fig.SI 3. Toy model - Autocorrelation a) - f) The purple arrow represents the velocity vector at time t_0, $\vec{v}(t_0)$, the green arrow the velocity vector at time t_0+t, $\vec{v}(t_0+t)$. For the sake of simplicity, we assume that they are both unitary vectors. **a)** $\vec{v}(t_0)$ and $\vec{v}(t_0+t)$ are parallel. Their scalar product is equal to 1, hence $c(t_0,t)=1$. **b)** $\vec{v}(t_0)$ and $\vec{v}(t_0+t)$ are orthogonal. Their scalar product is equal to 0, hence $c(t_0,t)=0$. **c)** $\vec{v}(t_0)$ and $\vec{v}(t_0+t)$ are anti-parallel. Their scalar product is equal to -1, hence $c(t_0,t)=-1$. **d) - f)** The purple circle represents the particle at time t_0, green circle at time t_0+t. **d)** At time $t=0$, and t equal to multiples of the revolution period T, $\vec{v}(t_0)=\vec{v}(t_0+t)$, hence $c(t_0,t)=1$. **e)** At time $t=T/4, 3T/4, \cdots, T/4+kT/2$, $\vec{v}(t_0)$ is orthogonal to $\vec{v}(t_0+t)$, hence $c(t_0,t)=0$. **f)** At time $t=T/2, 3T/2, \cdots, T/2+kT$, $\vec{v}(t_0)$ and $\vec{v}(t_0+t)$ are anti-parallel, hence $c(t_0,t)=-1$. **g)** The autocorrelation function of a particle moving with uniform circular motion, $\hat{C}(t)$, is a sinusoidal function, precisely $\hat{C}(t)=\cos(\omega t)$, with amplitude 1 (regardless of the radius of the circle along which the particle moves) and with the period equal to the revolution period of the particle.

2.1 Toy model

For the toy model introduced in Section1 of the SI, we can give an analytic form of the autocorrelation

$$c(t_0,t)=(\omega r)^2\cos(\omega t) \tag{12}$$

which is obtained by noting that:

$$\vec{v}(t_0)\cdot\vec{v}(t_0+t)=r^2\omega^2(\sin(\omega t_0)\sin(\omega t_0+t)+\cos(\omega t_0)\cos(\omega t_0+t)) \tag{13}$$

and by applying the subtraction formula for the cosine.

$c(t_0,t)$ does not depend on t_0, hence $C(t)=(\omega r)^2\cos(\omega t)$. The normalized correlation function, shown in Fig.SI3g, is then $\hat{C}(t)=\cos(\omega t)$, which is sinusoidal with amplitude 1 and period T.

2.2 Real data

When working with experimental data, time becomes a discrete variable defined by the camera frame rate, i.e. the frame rate (fps) fixes the minimum time step $\Delta t=1/fps$. Velocity vectors cannot be obtained by computing the derivative of the position vectors, but they are approximated.

From the reconstructed $3D$ trajectories of individual mosquitoes, $\vec{x}$, we compute individual velocities, $\vec{v}$, from two consecutive frames:

$$\vec{v}(t_j)=\frac{\vec{x}(t_{j+1})-\vec{x}(t_j)}{\Delta t} \tag{14}$$

where $t_j=j\Delta t$ denotes the instant of time corresponding to the j-th frame, $j=0,\cdots,J-1$ with J representing the last frame of the acquisition. Note that we are computing the velocity at frame j from the position at frame j and $j+1$, hence we cannot compute the velocity for the very last frame where trajectories are defined.

The autocorrelation function is defined in discrete time, as for the velocity vectors. The average over time defined in Eq.11 is computed as the following finite sum:

$$C(t_j) = \frac{1}{T_{max} - t_j} \sum_{k=0}^{J-1} c(t_k, t_j) \tag{15}$$

with $t_k = k\Delta t$, J is the last frame of the acquisition, and $T_{max} = J\Delta t$ is the time corresponding to the last frame.

It is worth noting that a value equal to 0 in the autocorrelation, may come from different situations. One possibility is that $c(t_0,t) = 0$ for each t_0, hence the average over t_0 gives $C(t) = 0$. This is the case at $t = T/4$, in the uniform circular motion described above. But there is also a second possibility, more likely to occur with real data: $c(t_0,t)$ are not null, but their average is equal to 0. In this latter case, the null value of the $C(t)$ has nothing to do with the orthogonality between $\vec{v}(t_0)$ and $\vec{v}_{t_0+t}$. $C(t)$ equal to 0 is then the evidence that there is not a typical value for the mutual orientation between $\vec{v}(t_0)$ and $\vec{v}(t_0+t)$, which are then uncorrelated. It is also important to note that for the $\hat{C}(t)$ to be equal to 1 or -1, all the $c(t_0,t)$ have to be equal to the normalization factor (all with positive or negative sign). This is a very particular condition, which occurs with the uniform circular motion for $t = k\pi$. But with real data it generally occurs only for $t = 0$, because of the normalization that actually imposes the condition $\hat{C}(0) = 1$.

In Fig.6 of the main text, we show autocorrelation functions of three individual mosquitoes. In order to check whether or not mosquitoes perform ring-like patterns in horizontal planes, we restricted the velocity autocorrelation function to the x and z components of the velocities, in such a way that $c(t_0,t)$ is defined as:

$$c(t_0,t) = v_x(t_0)v_x(t_0+t) + v_z(t_0)v_z(t_0+t) \tag{16}$$

These autocorrelation functions, restricted to the xz plane, clearly show a typical periodic trend with a period of about $1s$. The three plots are quite similar to each other, and are compatible with pseudo-circular motion. The amplitude of $C(t)$ decreases with time, while it would be constant and equal to 1 if the particle were moving in a perfect circle. This discrepancy is due to biological noise, which causes mosquitoes to alternate between circular and straight paths, rather than executing perfect circles, with variations in the radius of the circle and the period of revolution.

3 Single-sex male Swarm Data Table

Event label	generation	N	$NN(cm)$
20200923_ACQ1	4	52	7.7
20200923_ACQ2	4	76	7.7
20200923_ACQ3	4	113	6.8
20200923_ACQ4	4	95	6.9
20200928_ACQ1	5	183	6.9
20200928_ACQ2	5	208	5.9
20200928_ACQ3	5	206	6.6
20200928_ACQ4	5	27	10.0
20210525_ACQ1_S1	7	22	12.6
20210525_ACQ1_S2	7	18	11.9
20210525_ACQ2_S1	7	28	8.5
20210525_ACQ2_S2	7	25	7.3
20210525_ACQ3	7	53	7.6
20210525_ACQ4_S1	7	103	7.1
20210525_ACQ4_S2	7	28	12.0
20210525_ACQ5_S1	7	65	9.2
20210525_ACQ5_S2	7	175	6.9
20210525_ACQ6_S1	7	62	9.0
20210525_ACQ6_S2	7	139	8.2

Table SI 1. Single-sex male Swarm Data. Each row represents a different swarming event, denoted with a date, an acquisition number and in some cases, when more than one swarm was found in one acquisition, we add the suffix S to specify the corresponding swarm. N indicates the number of mosquitoes participating in the swarm, while NN indicates the mean nearest neighbour distance for the swarm as a whole.
